# BRAF/MEK inhibitors use for pediatric gliomas; real world experience from a resource-limited country

**DOI:** 10.3389/fonc.2024.1417484

**Published:** 2024-09-27

**Authors:** Dima Abu Laban, Abeer Alsharif, Maysa Al-Hussaini, Mouness Obeidat, Bayan Maraqa, Qasem Alzoubi, Awni Musharbash, Saad Jaddoua, Raed Ramlawi, Kawther Khaleifeh, Ahmad Kh. Ibrahimi, Nasim Sarhan, Eric Bouffet, Nisreen Amayiri

**Affiliations:** ^1^ Department of Diagnostic radiology, King Hussein Cancer Center, Amman, Jordan; ^2^ Department of Pharmacy, King Hussein Cancer Center, Amman, Jordan; ^3^ Department of Pathology, King Hussein Cancer Center, Amman, Jordan; ^4^ Department of Surgery, King Hussein Cancer Center, Amman, Jordan; ^5^ Department of Nursing, King Hussein Cancer Center, Amman, Jordan; ^6^ Department of Radiation Oncology, King Hussein Cancer Center, Amman, Jordan; ^7^ Division of Hematology/Oncology, The Hospital for Sick Children, Toronto, ON, Canada; ^8^ Department of Pediatrics, King Hussein Cancer Center, Amman, Jordan

**Keywords:** BRAF/MEK inhibitors, dabrafenib, trametinib, low-middle-income countries (LMIC), targeted therapy, glioma, off-label/compassionate

## Abstract

**Introduction:**

Most pediatric low-grade-gliomas (LGG) and some high-grade-gliomas (HGG) have alterations in the RAS/MAPK pathway. Promising high tumor response rates were achieved using BRAF/MEK inhibitors, however data on their use in low-middle-income-countries (LMICs) are limited.

**Methods:**

We retrospectively reviewed our Jordanian experience of using compassionate BRAF/MEK inhibitors in treating children with gliomas. We reviewed patients’ clinical characteristics, tumor response, and side effects.

**Results:**

Twenty patients (13 males, 7 females) were identified. Median age at diagnosis was 8.3 years (0.3-18.9years). There were fifteen LGGs, three HGGs and two grade-2 pleomorphic xanthoastrocytoma (PXA-2). Fifteen tumors were supratentorial, three posterior fossa/brainstem, one diffuse-glioneuronal tumor (DLGNT) and one spinal. Five tumors were metastatic. Except for one patient with neurofibromatosis, ten patients underwent partial resection and nine had biopsy. All patients, except three, received BRAF/MEK inhibitors after initial standard chemo/radiotherapy. Seven LGGs had BRAF-mutation, six had BRAF-fusion, and two were empirically treated (one neurofibromatosis and one DLGNT). Fourteen LGGs were treated with 1-4 chemotherapy regimens before BRAF/MEK inhibitors’ use; all had partial/stable response on targeted therapy at a median of 1.9 years (0.5-5.4years). Two patients with *BRAFv600E*-mutated/*CDKN2A* deleted PXA-2, had progression following resection, and experienced stable/partial response at 9 months of dabrafenib use. Two patients with HGGs had *BRAFv600E*-mutation, and one had an FGFR-mutation. All three patients with HGG had temporary stable/partial response, two with significant clinical improvement. At a median of 2.7 years (1.3-3.2years), all patients experienced tumor progression, and two died. Eight patients (40%) developed acneiform rash, three (15%) paronychia, and one had significant panniculitis and fatigue. Six patients (30%) needed dose-reduction. Nine patients had temporary drug interruptions [due to side effects (5) and drug shortage (4)]. Two patients who stopped trametinib due to side effects (significant acneiform rash/paronychia and intracranial bleeding) did not experience progression.

**Conclusions:**

Our experience with BRAF/MEK inhibitors’ use was positive achieving response in all LGGs and provided sustained response with good quality of life for patients with HGG. Cost effectiveness analyses and patients’ satisfaction comparisons with chemotherapy are needed to evaluate the routine use of these drugs in LMICs.

## Introduction

Gliomas are the most common pediatric CNS tumors with low-grade glioma (LGG) being more prevalent than high-grade glioma (HGG). LGGs are usually cured with gross tumor resection (GTR), however this is not achievable at every neuroaxis location, nor it is enough when the tumor is metastatic. The decision to treat or not incompletely resected or unresectable LGGs and with what modality depends on many factors including the child’s age, neurofibromatosis (NF1) status, size of the residual tumor, the anticipated neurological compromise with further tumor progression, and the availability of treatment modalities (chemotherapeutic agents or radiotherapy) (1). Several chemotherapeutic protocols (vincristine/carboplatin, vinblastine, TPCV) are considered as first, second and third lines of treatments for unresectable or progressing LGGs achieving a 5-year progression free survival (PFS) of 30-50% (2–4). While radiotherapy achieves higher PFS rates >70% (5, 6), its long-term neurocognitive and neuroendocrine side effects preclude its use as a frontline therapy in young children. While overall survival (OS) of patients with LGG is high (>80%) (2–4), PFS is low (<50%) highlighting the importance of preserving the best quality of life (QoL) for these children who may require multiple lines of treatment. In comparison, HGGs have poor prognosis (3year-OS < 30%) (7)despite surgery, radiotherapy, and chemotherapy, therefore maintaining a decent QoL during this short survival is integral.

Most LGG (>80%) harbor a driver alteration in the RAS/MAPK pathway signaling which makes this a plausible target for medical intervention (8). The type of this alteration plays a major role in the tumor trajectory, response to therapy and the risk of transformation to HGG. The presence of *BRAFv600E* mutation in a LGG (which occurs in 15-20%) was associated with a worse PFS and a higher risk of transformation to HGG even in the absence of radiotherapy (9). On the other hand, BRAF mutations are uncommon in pediatric HGGs (5-10%) (10) where the most frequent alteration is the *H3K27M* mutation (11). Integration of the molecular diagnosis with the histologic features is now required for several tumor types according to the WHO-CNS-5 classification (11). While this approach provides a more accurate diagnosis and a better understanding of the tumor’s behavior, it also helps in utilizing some targeted drugs for treatment. Several publications have demonstrated the efficacy of BRAF/MEK inhibitors in treating progressive LGGs (12–15) and HGGs (16–18) leading recently to the FDA approval of the dabrafenib and trametinib combination for the first line treatment of *BRAFv600E* mutated LGGs (19).

In a resource-limited setting, access to “new drugs” is challenging. These countries barely participate in international clinical trials and most families are not able to afford the high cost of these new drugs. On occasions, temporary access through off-label and compassionate drug access programs may be available to some institutions. This is not an ideal situation, however increasingly, off-label and compassionate use prescriptions are becoming common in the pediatric oncology world with the limited approved treatments for children and the scarce number of pediatric clinical trials (20, 21). There are very few publications on the use of compassionate targeted drugs in treating pediatric CNS gliomas in low-middle-income countries (LMICs) (22, 23).

Jordan is a LMIC according to the World Bank classification (24) with an estimated population of 10.3 million (including 37.7% are children aged 0-17 years old) (25). King Hussein Cancer center (KHCC), is the only cancer-dedicated hospital in Jordan to treat children and adults. Most children (> 80%) with CNS tumors are treated at KHCC. All Jordanians are insured through the Jordanian government for cancer therapy, while most non-Jordanians are covered through charities or self-paid.

In this study, we report on the compassionate use of dabrafenib and/or trametinib in pediatric patients with gliomas at KHCC. We demonstrated its feasibility, efficacy, and plausibility for the patients. In addition, this experience displayed the challenges encountered particularly in relation to the sustainability of access to these drugs.

## Methods

We retrospectively reviewed the medical charts of all children <18 years old at the time of diagnosis of gliomas at KHCC who received dabrafenib and/or trametinib before December 2023. The earliest child received therapy was in 2015. Targeted therapies were provided through a compassionate drug access program from Novartis. The decision to request and start the drugs was made by the multidisciplinary pediatric neuro-oncology team (MDT) and approved by the pharmacy and therapeutics committee at KHCC. We reviewed our patients’ clinical characteristics, tumor pathology and molecular alterations. We assessed the indication behind using dabrafenib/trametinib, drugs’ side effects and any clinical or radiological responses achieved.

Tumor diagnosis was extracted from the pathology reports issued by the KHCC neuropathologists. BRAF mutation was confirmed by immunohistochemistry (IHC), mutation analysis or TruSight next generation sequencing (NGS) (26). BRAF fusion was tested either by nanoString or NGS testing; both were performed at the laboratory of the Hospital for Sick Children (Sickkids) in Toronto. Not all gliomas were tested for molecular alterations. The decision to do so was based on the MDT discussions after weighing the likelihood of finding an alteration, the clinical condition of the patient, response of tumor to previous therapies (if previous treatment was given) and the expectations to have access to the targeted therapy. Once an alteration was found and compassionate access was available, the case was discussed again in the MDT to review if targeted therapy was needed immediately. This would be mostly in the context of tumor growth/progression despite previously administered chemotherapy and/or radiotherapy.

Tumor characteristics on MRI were reviewed for tumor location, presence or absence of metastasis, and response. GTR was considered if no residual tumor could be appreciated on the postoperative MRI, subtotal resection (STR) when a residual tumor is present, and a biopsy was considered if reported as such by the neurosurgeon. MRI scans just before and after the use of dabrafenib/trametinib were reviewed by the KHCC radiologist (D.A) according to the RANO criteria (27). These were reported as complete response (CR) in the absence of a residual tumor, partial response (PR) if the sum of the perpendicular diameter of the mass improved by 50% or more, stable disease (SD) if sum of the perpendicular diameter of the mass remained unchanged, improved by < 50% or increased by <25%. Progression was considered if the perpendicular diameter of the mass increased >25% or if new lesions appeared.

Drugs’ side effects that were suspected to be related to the use of dabrafenib/trametinib were extracted from the medical charts. A need for drug dose reduction, steroids use, or interruption/discontinuation of therapy was documented. For this study, parents, and children (older than 12-year-old) were asked to fill a one-time short questionnaire (**Supplementary Table S1**) on their opinion on the use of dabrafenib/trametinib; what they like, and dislike of this treatment option compared to chemotherapy (if it was previously prescribed). The questionnaire was administered between June and December 2023.

This study was approved by the Institutional Review Board at KHCC.

## Results

**Figure 1:**
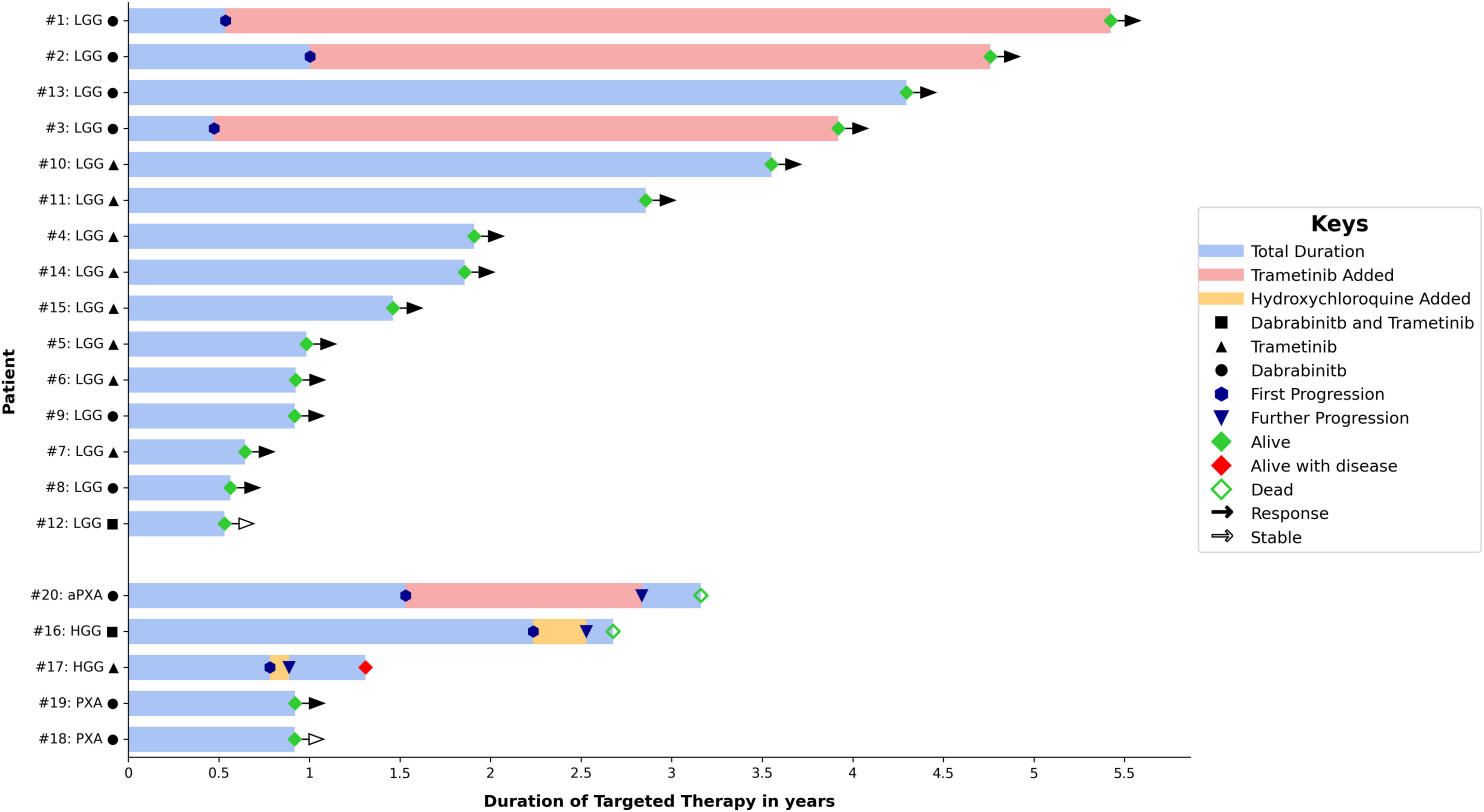
Change from baseline in tumor measurement and duration of response.

Twenty patients were identified, 13 males and 7 females (**Table 1**). The median age at diagnosis was 8.3 years (range, 0.3-18.9 years). The oldest patient (**Table 2**, #4) was originally treated for posterior fossa pilocytic astrocytoma (PA) with partial resection followed by vincristine and carboplatin. Then he was observed regularly with a stable residual tumor for 7 years. At 18.9 years, significant tumor progression upon transformation to glioblastoma was noted (**Supplementary Figure S1**). The retrospective analysis of the initial tumor identified a *BRAF V600E* mutation associated with *CDKN2A* deletion. There were 3 patients with HGG, two with pleomorphic xanthoastrocytoma (PXA, WHO grade 2) and 15 with LGG. Fifteen tumors were supratentorial, three were in the posterior fossa/brainstem, one diffuse leptomeningeal glioneuronal tumor (DLGNT) and one primary spinal LGG. Five tumors were metastatic at time of initiation of the targeted therapy: two HGG, one DLGNT, one posterior fossa PA and one suprasellar desmoplastic infantile astrocytoma (DIA). Except for one patient with NF1, all patients had tissue proven diagnosis. Ten patients underwent STR and nine had tumor biopsy. All patients, except three, received dabrafenib and/or trametinib after the standard treatment protocol (chemotherapy with/without radiotherapy). Summary of patients’ and tumors’ characteristics, treatment received, response to targeted therapy and duration are demonstrated in **Table 1** and **Figure 1**.

**Table 1:** Summary of patients’ and tumors’ characteristics, treatment received and response to targeted therapy.

Diagnosis	LGG	PXA	HGG
Number of patients	15	2	3
Molecular tumor characteristics			
BRAF fusion	6	0	0
BRAF mutation	7	2	2
CDKN2A deletion	NA	2	1 (2 NA)
FGFR mutation	0	0	1
Empirical therapy	2	0	0
Tumor metastasis at start of targeted therapy	3	0	2
Treatment received			
Dabrafenib alone	6 (then 3 had trametinib added)	2	1 (then trametinib was added)
Trametinib alone	8	0	1
Combination	1	0	1
Initial radiological response	10 PR, 5 SD	1 PR, 1 SD	3 PR
Progression	0	0	3
Median follow up	1.9 years (range, 0.5-5.4 )	9 months	2.7 years (range, 1.3-3.2 )
Death	0	0	2

NA, not available; PR, partial response; SD, stable disease.

**Table 2:** Characteristics of patients with low grade glioma and their treatment.

#	Diagnosis and Molecular alteration	Initial treatment	Tumor status before targeted therapy	Targeted therapy / Response & duration (months)	Further therapy	Response	Progression	Total duration of targeted therapy (year)	Patient outcome /duration of survival (year)
1	Suprasellar PA, *BRAFv600E* mutation (IHC)	VCR/Carboplatin (15 cycles) then vinblastine (51 weeks)	Local progression	Dabrafenib / progression (6)	Trametinib was added	Partial response	No	5.4	Alive / 12.9
2	Suprasellar PA, *BRAFv600E* mutation (IHC)	VCR/Carboplatin (14 cycles) then vinblastine (42 weeks) then TPCV (8 cycles)	Local progression with visual decline	Dabrafenib /stable (12)	Trametinib was added to control side effects	Partial response & resolution of panniculitis/ fatigue	No	4.8	Alive /9.5
3	Suprasellar PA, *BRAFv600E* mutation (IHC)	VCR/Carboplatin (15 cycles) then vinblastine (70 weeks)	Local progression with visual decline	Dabrafenib/ progression (6)	Trametinib was added	Partial response	No	3.9	Alive /11.1
4	Suprasellar PA, *KIAA1549_Ex15-BRAF_Ex9* fusion (NGS)	VCR/Carboplatin (15 cycles) then vinblastine (70 weeks) then vinorelbine (7 cycles)	Local progression with visual decline	Trametinib/partial response			No	1.9	Alive /7.3
5	Suprasellar PA, *KIAA1549::BRAF* fusion (NGS)	VCR/Carboplatin (13 cycles) then vinblastine (10 weeks)	Local progression with risk on residual vision	Trametinib/ stable			No	1	Alive /4.6
6	Suprasellar PA, *KIAA1549(exon15)::BRAF(exon9)* fusion (NGS)	VCR/Carboplatin (7 cycles)	Local and metastatic progression with diencephalic syndrome	Trametinib/ partial response with weight gain			No	0.9	Alive /1.4
7	Suprasellar PA, *KIAA1549(Ex16)::BRAF(Ex09)* fusion	VCR/Carboplatin (12 cycles), then vinblastine (68 weeks) then TPCV (7 cycles)	Local and metastatic progression with risk on residual vision	Trametinib/ partial response			No	0.6	Alive /12.1
8	Suprasellar ganglioglioma, *BRAF V600E* mutation, *CDKN2A*- no loss of expression (NGS)	VCR/Carboplatin (2 cycles)	Symptomatic local progression	Dabrafenib/ partial response with significant clinical improvement			No	0.6	Alive /0.8
9	Suprasellar metastatic DIA, *BRAFv600E* mutation (IHC)	---	Developed ascites following ventriculo-peritoneal shunt insertion	Dabrafenib /partial response with resolution of ascites without VA insertion			No	0.9	Alive /1
10	Suprasellar and thalamic/basal ganglia PA, (NF1)	VCR/Carboplatin (7 cycles), then surgery then vinblastine (57 weeks)	Local progression	Trametinib/ Stable disease (stopped therapy later)			No (off trametinib 4 months)	3.6	Alive /8.9
11	Metastatic posterior fossa PA, *KIAA1549_Ex15-BRAF_Ex9* fusion (NGS)	VCR/Carboplatin (6 cycles) Then vinblastine (52weeks) then TPCV (5cycles) then vinorelbine (17 cycles) and surgery	Symptomatic local and metastatic progression with significant pains	Trametinib/ stable disease (stopped therapy later)			No (off trametinib 9 months)	2.9	Alive /11.7
12	Frontotemporal DIG, *BRAFp.G469A* (NGS)	Baby POG protocol (6 cycles)	Variable tumor growth and developed ascites	Dabrafenib and Trametinib/ stable disease with resolution of ascites without VA insertion			No	0.5	Alive /2.1
13	Cervico-medullary ganglioglioma, *BRAFv600E* mutation (PCR)	VCR/Carboplatin (7 cycles), then surgery, then vinblastine (50 weeks)	Symptomatic local progression	Dabrafenib/ partial response			No	4.3	Alive /8.7
14	DLGNT, tumor RNA quantity not enough for NGS	VCR/Carboplatin (3cycles), and focal spinal radiotherapy (cord compression)	Intracranial metastatic progression	Trametinib/ Partial response in brain, stable in spine			No	1.9	Alive /2.1
15	Spinal fibrillary astrocytoma, *KIAA1549_Ex15-BRAF_Ex9* fusion (NGS)	Vinblastine (52weeks) then VCR/Carboplatin (10 cycles)	Symptomatic local progression	Trametinib/ stable disease			No	1.5	Alive /11.5

DIA, desmoplastic infantile astrocytoma; DIG, desmoplastic infantile ganglioglioma; DLGNT, diffuse leptomeningeal glioneuronal tumors; F, female; IHC, immunohistochemistry; M, male; NF1, neurofibromatosis type 1; NGS, next generation sequencing; PA, pilocytic astrocytoma; PCR, polymerase chain reaction; POG, pediatric oncology group; TPCV, thioguanine/ procarbazine/ lomustine/ vincristine; VA; ventriculo-atrial shunt; VCR, vincristine.

### Patients with LGG

**Figure 2:**
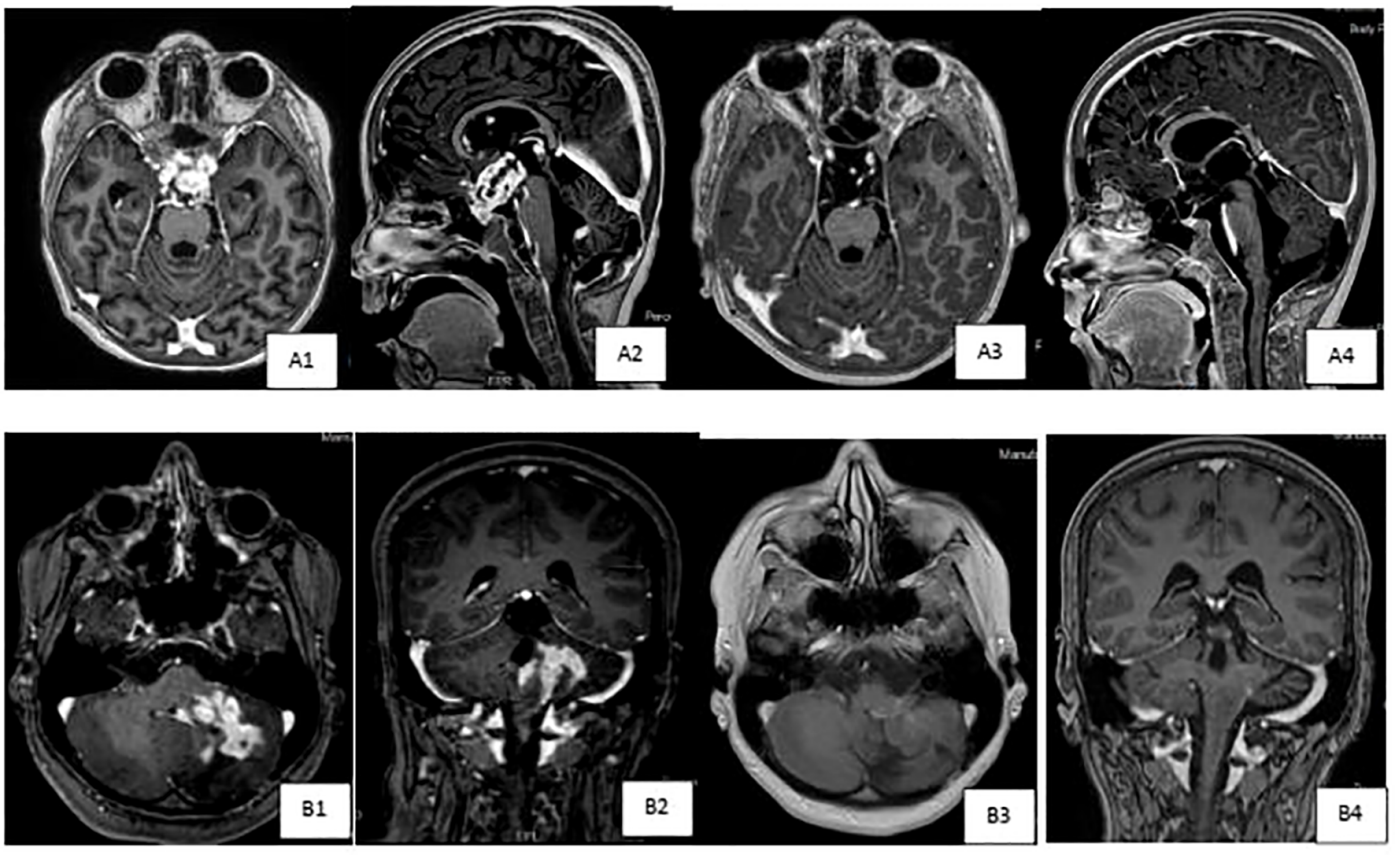
Brain MRI scans demonstrating tumor response to targeted therapy in two patients with low grade gliomas. **(A)** Axialand sagittal T1-weighted post IV contrast brain images of patient # 14 with diffuse leptomeningeal glioneuronal tumor (DLGNT),demonstrating pretreatment (A1& A2) large contrast enhancing mass in the suprasellar cistern,inseparable from the optic chiasm,extending to the floor of the third ventricle. Subependymal,intraventricular enhancing nodules are also noted, seen onlower row images. Marked interval tumor response {A3 & A4) with resolution of cortrast enhancement with almost resolution of previously seen subependymal enhancing nodules,currently much smaller and non­enhancing, as seen in upper row images. **(B)** Axialand coronal T1-weighted post IV contrast brain images of patient # 13 with cervico-medullary gangiloglioma,demonstrating pretreatment (B1 & B2) heterogeneous contrast enhancingmass in the left cerebellar hemisphere,with the involvement of the brain stem,particularly l eft hemi medulla,and leptomeningealenhancement extending to the left foramen of Luschka.Marked interval tumor response (B3 & B4) in the tumoral component within theleft cerebellar hemisphere, with almost resolution of mass like contrast enhancement,development of leukomalacia, improvement in the expansion of the left hemi medulla and contrast enhancement.

We identified 15 patients with LGG (**Table 2**); nine males and six females at a median age of 5.4 years (range, 0.3- 13.1 years) at diagnosis. Ten patients had optic hypothalamic pathway gliomas (OPG). Three tumors were metastatic. Ten patients underwent tumor biopsy and five had STR. Nine tumors were PA, two DIA/DIG, two gangliogliomas, one fibrillary astrocytoma, and one DLGNT. Seven tumors had BRAF mutation (one was a rare mutation: *BRAFp.G469A*), six had BRAF fusion, and two were empirically treated; one (#10) had NF1 and one (#14) with DLGNT had small tumor biopsy insufficient for NGS testing. Tumors with BRAF mutation were treated with dabrafenib and trametinib was added after tumor progression, while tumors with BRAF fusion, NF1 or DLGNT were treated with trametinib. Six patients were started on dabrafenib alone and later trametinib was added in three of them; two due to tumor progression and one to help control the side effects. After adding trametinib, this patient (#2) could be weaned off opioids and steroids that were used to control his panniculitis and fatigue. Eight patients were initially started on trametinib, and one patient (#12) was started on the combination of dabrafenib and trametinib due to his rare mutation (*BRAFp.G469A*). All patients except one used dabrafenib/trametinib after tumor progression following chemotherapy use. This one patient (#9), who was previously reported, underwent a ventriculoperitoneal shunt insertion and biopsy of his metastatic DIA, and later developed ascites. Dabrafenib achieved significant tumor response and ascites resolved without a need for permanent shunt diversion. All patients, except two (#10 & #11), are continuing treatment. All tumors showed SD or PR at a median follow up of 1.9 years (range, 0.5-5.4 years) from starting dabrafenib/trametinib. **Figure 2** demonstrates the tumor response to targeted therapy in two patients with LGG. The two patients who stopped trametinib (#10 & #11) had no tumor progression on follow-up MRI scans at 4 and 9 months, respectively.

### Patients with PXA and HGG

Two patients with supratentorial PXA-2 underwent STR and GTR, respectively (**Table 3**). On histology, their tumor exhibited high risk features. Both patients had tumor progression within 3 months. Because further surgical resection was felt to achieve less than GTR, and to avoid giving radiotherapy, a trial of medical therapy was felt reasonable. Both had *BRAFv600E* mutation and *CDKN2A* deletion. Dabrafenib was started and during the first 9 months SD and PR were achieved respectively.

**Table 3:** Characteristics of patients with pleomorphic xanthoastrocytoma and high grade glioma and their treatment.

#	Diagnosis/ Molecular alteration	Initial treatment	Tumor status before targeted therapy	Targeted therapy	Response & duration (months)	More therapy	Response /duration	Progression	Total duration of targeted therapy (year)	Patient outcome /duration of survival (year)
1	Tempero-parietal PXA, *BRAFv600E* mutation (FISH) and CDKN2A deletion	STR	Asymptomatic local progression	Dabrafenib	Stable	----	---	No	0.9	Alive / 1.3
2	Tempero-parietal PXA, *BRAFv600E* mutation (FISH) and CDKN2A deletion	GTR	Asymptomatic local progression	Dabrafenib	Partial response	----	---	No	0.9	Alive / 1.2
3	Tempero-parietal aPXA *BRAFv600E* mutation (IHC)	STR/focal rads with TMZ then TMZ 10 cycles then STR followed by Procarbazine /CCNU/ Vincristine (1 cycle)	Symptomatic local and leptomeningeal metastasis	Dabrafenib	Partial response (15) with significant clinical improvement	Partial resection/ added Trametinib	Stable (15 months)	Local and lepto-meningeal metastasis	3.2	Dead /6.7
4	Posterior fossa high grade glioma *BRAFv600E* mutation (IHC) and CDKN2A deletion *	STR/focal rads with TMZ then TMZ (7 cycles)	Symptomatic local progression	Dabrafenib and Trametinib	Partial response (24)	HQC was added upon lepto-meningeal progression	Partial response (2 months)	Lepto-meningeal metastasis	2.7	Dead / 3.7
5	Metastatic thalamic DMG, *H3K27M* altered NGS: *FGFR1p.K656E* and *PTENp.F341V*	PR/WBR with TMZ then TMZ 7 cycles	Asymptomatic leptomeningeal metastasis	CSI then Trametinib	Stable (9)	HQC was added upon lepto-meningeal progression then stopped in 2 months due to limited response	Progression	Lepto-meningeal metastasis	1.3	Alive with disease / 2.4

aPXA, anaplastic pleomorphic xanthoastrocytoma; CSI, craniospinal radiotherapy; DMG, diffuse midline glioma; F, female; FISH, Fluorescence in situ hybridization; GTR, gross tumor resection; HCQ, hydroxychloroquine; IHC, immunohistochemistry; M, male; PXA, pleomorphic xanthoastrocytoma; STR, subtotal tumor resection; TMZ, temozolomide.

*This patient was originally treated for posterior fossa pilocytic astrocytoma with partial resection followed by vincristine and carboplatin. Then he was observed with regular MRI scans showing stable residual tumor for 7 years before his tumor transformed to high grade glioma.

Three patients had HGG (**Table 3**); one had multiple recurrent BRAF mutated aPXA [#3, previously published (23)] was treated with dabrafenib then trametinib was added upon progression, one had posterior fossa BRAF mutant PA transformed to HGG after 7 years without prior radiotherapy use and was started on combined dabrafenib and trametinib, and the third had K27M altered HGG with *FGFR1p.K656E* and ependymal metastatic lesions who received trametinib and still alive with disease. All tumors underwent STR followed by radiotherapy and temozolomide, then upon further tumor progression they received dabrafenib and/or trametinib. In addition to the radiological response, two patients (#3 & 4) had significant symptomatic improvement. In two patients, hydroxychloroquine was tried to overcome the drug resistance; this was temporarily successful in one patient. With a median of 2.7 years (range, 1.3-3.2 years) from starting dabrafenib and/or trametinib, all tumors progressed, and two patients died.

### BRAF/MEK inhibitors side effects

Eight patients (40%) developed acneiform rash; six were on trametinib alone. Three patients (15%) developed paronychia, and one had panniculitis (needing opioids and systemic steroid use) with fatigue. Six patients (30%) needed dose reduction in addition to the supportive measures. Panniculitis and fatigue resolved with addition of trametinib in patient (#2 in **Table 2**). Ophthalmic and cardiac toxicities were not reported on our regular assessments. One patient (#11 in **Table 2**) with a difficult to control metastatic LGG, stopped trametinib after 2.2 years despite significant clinical response (became off multiple analgesics including opioids). She had repeated acneiform rash and significant paronychia needing multiple surgical debridement despite the medical care and drug interruptions. Nine months off trametinib, she was asymptomatic with no evidence of radiological tumor progression. One patient (#10 in **Table 2**) developed significant intracranial bleeding and trametinib was held. Four months later, his tumor did not re-grow. Nine patients had temporary drug interruptions: five due to drug-related side-effects and four due to periods of drug shortage. Three patients developed significant neurological symptoms coinciding with radiological tumor progression within 3 weeks of drug interruption.

### Parents and children’s opinions on using BRAF/MEK inhibitors

Eleven of 17 parents of patients with PXA or LGG answered the questionnaire (**Supplementary Table S1**) in addition to 5 of their children. Children had similar responses to their parents. Except for the patient who stopped trametinib due to side effects (#11 in **Table 2**), all others were very satisfied with the drugs and felt they were better than chemotherapy. They mainly liked the oral route of these drugs, less frequent hospital visits, the minimal hematological toxicity and lack of hair loss. They disliked the dermatological side effects, particularly those patients who had severe symptoms, and the drugs’ risks on the heart and retina. The risk of tumor progression with drug interruptions and the need to continue these drugs for long time was of a significant concern to the families.

## Discussion

We report for the first time on a series of children with gliomas treated with BRAF/MEK inhibitors in a resource-limited country. The compassionate drug access program allowed us to prescribe these drugs and achieve an excellent tumor control in LGGs and a temporary prolonged control in HGGs. Though most families were very satisfied using these new drugs, there are several challenges encountered.

Treating pediatric LGGs is an art that requires to balance tumor control with the treatment’s side effects. The discovery of the molecular landscape of pediatric LGGs and the integral role of the RAS/MAPK pathway signaling in tumorigenesis led to the introduction of BRAF/MEK inhibitors in their management. Many case series demonstrated their efficacy in the recurrent setting achieving reasonable tumor control with a favorable side effects’ profile. This triggered a still ongoing debate as whether targeted therapies should replace chemotherapy (28). A recently published phase II trial (29) on 110 children with *BRAFv600E-*mutated LGG randomized in a 2:1 ratio to receive dabrafenib and trametinib or standard chemotherapy (carboplatin and vincristine), led to the FDA approval of this combination as a frontline therapy (19). In this trial, and at a median follow-up of 18.9 months, overall tumor response occurred in 47% of children treated with targeted therapy compared to 11% for those given chemotherapy, with observed clinical benefit of 86% and 46% respectively. This resulted in a significantly longer median PFS in the dabrafenib/trametinib arm (20.1 months) compared to 7.4 months in the chemotherapy arm. Currently, the type II RAF inhibitor tovorafenib, is being investigated in a randomized phase 3 trial (30) as a frontline therapy compared to standard chemotherapy in children with BRAF-altered LGG. Type II RAF inhibitors result in tumor response regardless of the BRAF alteration type (mutation or fusion) without a risk of paradoxical activation.

In comparison, the outcome of pediatric HGG is significantly lower despite surgery, radiotherapy, and chemotherapy. *BRAFv600E-*mutated HGGs are a clinically distinct subtype, and most are secondary to transformed LGGs (10). Nobre et al (31) reported on eleven HGGs previously received radio-chemotherapy; four responded to targeted therapy (36%) with all but one tumor progressed in 18 months. Forty-one children with relapsed/refractory *BRAFV600E*-mutated HGG received combined dabrafenib and trametinib in a phase II trial (17) had overall response rate of 56% with a median duration of response of 22.2 months. At a median follow-up of 25.1 months, 51% of patients remained on treatment. This is exceptional in recurrent HGGs which rarely respond to chemotherapy resulting in OS of only few months. This raises the question of whether upfront use of BRAF/MEK inhibitors (32–34) is superior in children with HGGs to optimize their management and try to delay radiotherapy use with its deleterious neurocognitive side effects. One of our patients (#5, **Table 3**) had the unique entity of K27M altered HGG with FGFR1 mutation. His tumor response to trametinib and prolonged survival despite disease progression was previously described in the literature (35).

The use of dabrafenib/trametinib in our setting was encouraging. All gliomas showed tumor control, and though it was temporary in HGGs the duration was of the longest reported (1.3-3.2 years). Importantly, many patients experienced significant control of their symptoms; two children experienced dramatic improvement in their neurological function and were able to practice normal daily activities (**Table 1** patient # 8 & **Table 3** patient # 3), two patients were spared from a CSF diversion procedure for their ascites (**Table 1** patients # 9 &12) (36), one patient with significant sleep apnea became off night BiPap (**Table 1** patient #13), one patient became off pain control medications including opioids (**Table 1** patient #11), and one child with diencephalic syndrome gained weight (**Table 1** patient #6). These symptoms were not previously controlled despite the use of multiple lines of chemotherapy. We would argue whether the earlier introduction of dabrafenib/trametinib, with their rapid tumor response, would have saved some patients from the morbidities of recurrent tumor progressions, particularly on vision, and resulted in a better overall functional outcome. None of our patients with LGG had visual decline while using dabrafenib/trametinib, but several patients had dropping vision with previous tumor progressions. While we did not easily have the option of upfront use of dabrafenib/trametinib through the compassionate drug access program, it is clearly an FDA approved indication now for BRAF-mutated LGGs. This further supports the opinion that every CNS tumor should be tested molecularly as this can make a huge impact on the child’s management and outcome.

Our experience echoes the published data on the side effects’ profile of dabrafenib/trametinib. While most side effects are dermatological, mild, and manageable (17, 29) they can be very distressing to the patients particularly the adolescents. Meticulous skin care is needed to help control these side effects which can be very demanding and challenging to the patients. Emollients and sunscreens were regularly prescribed to our patients and most reported compliance using them. One patient (**Table 1**, #11), and despite the great control of her neuropathic pains, she could not tolerate the recurrent paronychia and acneiform rash. She eventually stopped trametinib despite her awareness of the risk of rebound and the possible need for radiotherapy. This is a well reported risk when stopping the targeted therapies (37). Fortunately, her tumor is still under control 9 months after discontinuation of treatment. Recently, experts from Canada developed a consensus algorithm for discontinuation of targeted therapies in children with *BRAFV600E* gliomas (38). One patient (**Table 1**, #10) developed significant intracranial bleeding while on trametinib. This rare event was previously reported in the literature (39). We did not notice cardiac dysfunctions or ophthalmic side effects in our cohort despite regular assessments. These risks were one of major drawbacks of using targeted therapies according to the families. In addition, the uncertainty on the duration of using these drugs, and the high risk of rebound tumor growth with drug interruptions were stressful to the families. This is still a medical challenge. There are anecdotal data on successful rechallenge after stopping BRAF inhibitors (31), or shifting to a selective BRAF inhibitor (40), or combining it with chemotherapy. Despite these risks, most of our patients preferred the use of targeted therapies over chemotherapy.

With the use of the compassionate drug access program, we provided new targeted drugs to our patients however this is not without a challenge. We had times with drugs interruptions related to drug importation and during the COVID era. This route of drug access is used globally particularly in children with cancer where there are limited drug approvals or clinical trials access (20). It may be more “justified” in a LMIC setting where access to new drugs will take long time, if ever. The high cost of the targeted drugs is a challenge for routine clinical use even after the accumulating evidence of efficacy in the literature. We are now working on a cost effectiveness analysis and specific indications to use dabrafenib/trametinib at KHCC after closure of the compassionate drug access program in Jordan following the FDA approval of the combination of trametinib and dabrafenib for pediatric patients with BRAF mutated LGGs in March 2023. It is important as well to consider the participation of LMICs in international clinical trials of new targeted medications. Most of these drugs are orally administered and need less frequent monitoring which makes the idea of using them is more plausible in a resource-limited setting. This hopefully would result in less abandonment of therapy or a need to use alternative choices with shorter duration of therapy, like radiotherapy, with its detrimental neurocognitive side-effects particularly on young children. In addition, most targeted drugs act rapidly which help decrease the morbidities associated with tumor growth (e.g. visual loss or neurological deficits) which are more difficult to “tolerate” in a resource-limited setting. On the other hand, inclusion of LMICs in the international clinical trials will help advance the whole health system in these countries.

The present study is limited by the fact it is a retrospective review of a single center experience in a resource-limited setting. KHCC is a relatively advanced center for a LMIC and has excellent infrastructure and trained staff. Furthermore, KHCC has a long-standing twinning program with SickKids hospital. This has contributed to facilitate the interaction with the team involved in the Novartis compassionate program, to build a strong relationship with this team and to be granted approvals for compassionate use for this entire cohort of patients. This makes our experience unique, as reports on targeted treatment in children with brain tumors in LMICs remains anecdotal (22). The response rate observed in our experience appears to be higher than in clinical trials of targeted therapies (29). This may be related to a selection bias in our MDT. However, discrepancies between institutional evaluation and central reviews were noted in several trials (29, 41), with higher response rates reported by investigators. Capturing toxicity data was limited by the retrospective nature of this review and the toxicity may appear lower than in prospective trials of targeted treatments. However, only significant side effects were captured particularly those resulted in dose reductions or interruptions. The positive insight provided by the parents and children on using dabrafenib/trametinib is encouraging and rarely documented in LMICs.

In conclusion, our experience demonstrates the feasibility of using new targeted drugs in a resource-limited setting and the effectiveness in achieving good tumor control with excellent patients’ satisfaction. Questions remain to be answered regarding the duration of using these drugs and their long-term toxicity in children. The current ethical challenge facing LMICs is to balance the affordability of using these drugs in routine clinical practice. Moving targeted drugs to the frontline can save children several morbidities and be more cost effective on the long-term even in a resource-limited setting. Well-designed global studies that combine patients’ reported outcome, families’ perspective, tumor response and cost effectiveness are needed.

## Data Availability

The original contributions presented in the study are included in the article/**Supplementary Material**. Further inquiries can be directed to the corresponding author/s.
