# Supplementary material for: BRAF/MEK inhibitors use for pediatric gliomas; real world experience from a resource-limited country
Source: Front Oncol. 2024 Sep 27;14:1417484. doi: 10.3389/fonc.2024.1417484 (PMC11466720; doi:10.3389/fonc.2024.1417484)
Supplement: Supplementary file 1 [file DataSheet1.pdf]

Question	Answer
How long it has been since you (your son/daughter) started using the targeted drugs?	
Are you satisfied of using targeted drugs?	
What are the things you **like** about targeted drugs (circle all what applies):	
1- Oral use	
2- No need for repeated IV sticks	
3- No major drop in neutrophil counts	
4- Less hospital visits	
5- Others (mention)	
What are the things you **dislike** about targeted drugs (circle all what applies):	
1- Needing to fast before and after having the drugs	
2- Skin and nail side effects	
3- Risks on heart	
4- Risks on retina	
5- Not knowing when the drugs will stop	
6- Others (mtnion)	
What is the most thing you **hate about targeted drugs?**	
What is the most thing you **hate about chemotherapy** (if previously given)?	
Compared to chemotherapy (if previously used), do you think targeted drugs are better or worse? And why?	
Do you think the tumor responded better to chemotherapy or to targeted drugs?	

Table 1 supplementary: Questions asked in the parents' /patients' questionnaire:
