# Supplementary figures and images for: BRAF/MEK inhibitors use for pediatric gliomas; real world experience from a resource-limited country
Source: Front Oncol. 2024 Sep 27;14:1417484. doi: 10.3389/fonc.2024.1417484 (PMC11466720; doi:10.3389/fonc.2024.1417484)

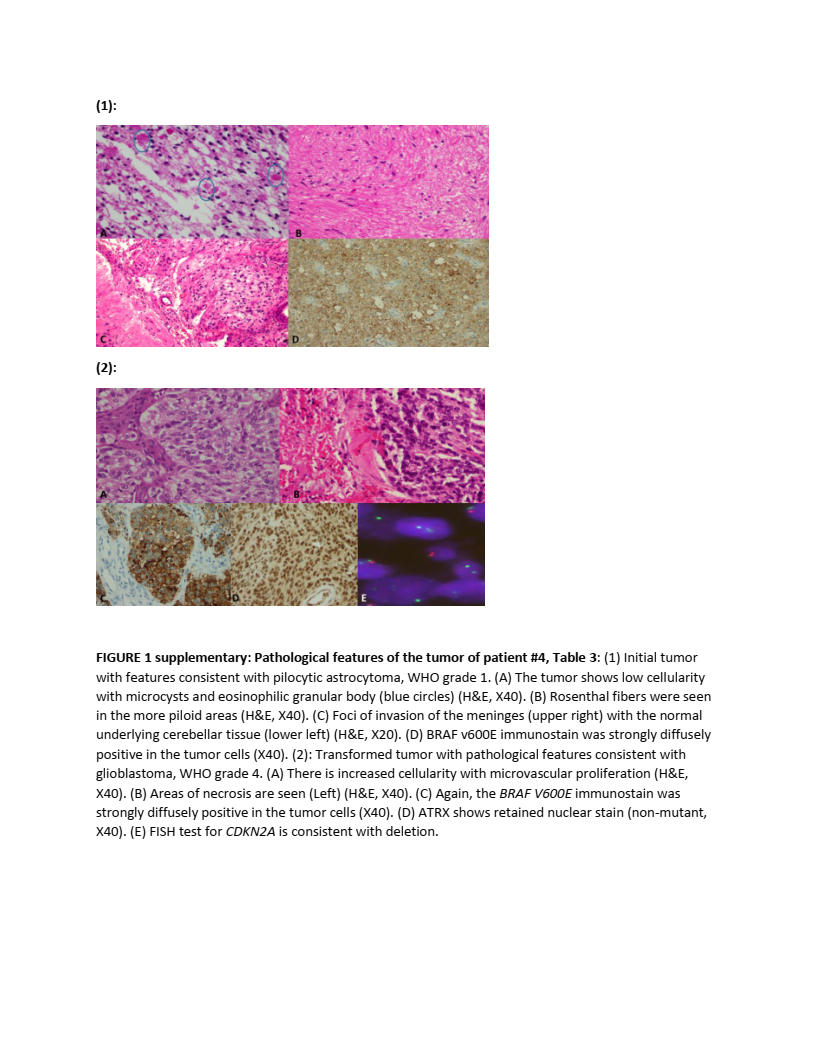

Supplement: Supplementary file 2 [file Image1.jpeg]
